# EvoApneaFormer: an IoT and prognostic evolutionary deep learning-based framework for real-time multi-event sleep apnea disorder detection and remote monitoring

**DOI:** 10.3389/fbioe.2026.1755071

**Published:** 2026-06-11

**Authors:** Abdullah, Zulaikha Fatima, José Luis Oropeza Rodríguez, Osvaldo Espinosa Sosa

**Affiliations:** 1 Center for Computing Research, Instituto Politécnico Nacional, Mexico City, Mexico; 2 Department of Computer Science, Bahria University Lahore Campus, Lahore, Pakistan; 3 Department of Allied Health Science, Superior University, Lahore, Pakistan

**Keywords:** edge AI for clinical diagnostics, edge computing in healthcare, EvoApneaFormer, IoT-enabled healthcare, low-power embedded systems, neuroevolutionary optimization, polysomnography alternatives, remote patient monitoring

## Abstract

**Introduction:**

Sleep apnea is a prevalent, yet underdiagnosed, disorder associated with cardiovascular disease, hypertension, and cognitive decline. Although polysomnography (PSG) is the diagnostic gold standard, its high cost > $6,000 per unit, invasiveness, and limited availability hinder large-scale screening.

**Methods:**

To overcome these barriers, we propose ApneaSense, a non-intrusive IoT-enabled diagnostic framework powered by EvoApneaFormer, an evolutionary transformer-based deep learning model for temporal bio-signal fusion and adaptive learning. EvoApneaFormer combines dynamic self-attention with neuroevolutionary optimization, enabling superior convergence, noise resilience, and generalization across heterogeneous signals. ApneaSense processes synchronized ECG, SpO2, respiratory effort, and motion data on low-power Raspberry Pi 4 hardware <2W using TensorFlow Lite quantization, with a companion Flutter app for real-time visualization, 72-h offline operation, and secure HL7/FHIR telehealth synchronization. Clinical validation on a hybrid dataset, the UCD Sleep Apnea Database (n = 25) and the ApneaSense Clinical Dataset (n = 61), with stratified patient-level splitting.

**Results:**

On the held-out test cohort (n = 17), EvoApneaFormer achieved 99.98% accuracy, 99.91% precision, 99.95% recall, a 99.93% F1-score, and a 0.999 macro-AUC. Six apnea classes, namely, normal, obstructive, central, mixed, hypopnea, and respiratory effort-related arousal (RERA), were each identified with ≥99.8% accuracy. Real-world trials confirmed robustness in both clinical 99.8% and home 96.9% settings, even under motion artifacts and signal dropouts. SHAP-based interpretability and uncertainty quantification were deemed actionable by 82% of clinicians.

**Discussion:**

Cost-effectiveness analysis indicated break-even in 6.2 months at $5,200/QALY. Compared to wearable oximeters, ApneaSense suggested a 9.98-point accuracy gain at one-third the cost per test, representing a paradigm shift in edge-based respiratory diagnostics for scalable and personalized sleep apnea monitoring in underserved communities.

## 1 Introduction

Sleep is a vital physiological process, essential for metabolic regulation, immune function, cognitive performance, and cardiovascular stability (Alimova et al., 2025). Humans spend nearly one-third of their lives asleep, and disruptions in sleep quality or duration have been linked to a wide spectrum of acute and chronic health conditions (Ortiz et al., 2025). Among these, sleep apnea encompassing obstructive sleep apnea (OSA) and central sleep apnea (CSA) is one of the most common, yet persistently underdiagnosed, sleep disorders (Pevernagie et al., 2025). Recent estimates from the World Health Organization in 2024 suggested that more than 1 billion individuals globally are affected by OSA, with up to 80%–90% remaining undiagnosed, especially in low- and middle-income regions (Iannella et al., 2025; Zhai et al., 2025). OSA is marked by recurrent upper airway obstructions during sleep, leading to intermittent hypoxia, sympathetic overactivation, and fragmented sleep architecture (Kaštelan et al., 2025; Birză et al., 2025). These disruptions are associated with heightened risks of cardiovascular disease, arrhythmia, hypertension, stroke, insulin resistance, and cognitive decline (Siddique et al., 2024; Fatima et al., 2025). In contrast, CSA results from impaired neural control of respiratory muscles, more commonly observed in older adults or patients with neurological and cardiac conditions. Both OSA and CSA degrade sleep quality, impair daytime functioning, and elevate long-term morbidity and mortality (Lee et al., 2025).

Considering these challenges, there is an urgent need for scalable, explainable, and cost-effective diagnostic alternatives that can operate in real-world, nonclinical settings without compromising accuracy (Haque et al., 2025; Hasan et al., 2025). Traditional machine learning approaches have been applied to simplified datasets derived from polysomnography (PSG) signals, yet they often lack the capacity to model complex temporal dynamics and multimodal interactions inherent in physiological data (Yang et al., 2025). Deep learning models, particularly convolutional and recurrent architectures, have shown promise in automatic feature extraction and pattern recognition from bio-signals (Saurabh and Gupta, 2024; Sahoo et al., 2024). Nevertheless, these models are frequently developed in siloed domains, evaluated on constrained datasets, and offer limited interpretability, posing significant barriers to clinical adoption (Medjahed and Boukhatem, 2025). Furthermore, few models consider the full diversity of apnea subtypes or generalize across heterogeneous populations. A cross-domain, explainable framework that integrates multimodal signals, adapts to individual variability, and provides clinically meaningful insights would mark a significant advancement in sleep medicine (Zhou et al., 2025; Woerner et al., 2025).

Despite its clinical importance, the diagnosis of sleep apnea continues to rely primarily on overnight PSG, a hospital-based, multi-channel recording involving EEG, ECG, EMG, airflow, and oxygen saturation (Kim et al., 2025). PSG is time-consuming, costly, typically exceeding $6,000 per unit, and requires trained technicians for administration and interpretation. Its intrusive setup can disrupt natural sleep behavior, further limiting diagnostic accuracy (Stefanos et al., 2025). Consequently, PSG is ill-suited for early screening or scalable deployment in underserved or rural communities. Advances in wearable biosensors, edge AI, and telehealth have opened new possibilities for real-time, low-cost, and non-intrusive sleep monitoring. However, most existing academic and commercial solutions suffer from critical limitations: (i) responsiveness and data privacy, (ii) lack of interpretability and clinical trustworthiness, and (iii) inadequate adaptation to inter-patient physiological variability (Zhang et al., 2025).

To address these critical gaps, we present ApneaSense, a comprehensive and deployable system for real-time sleep apnea detection and prognosis. Central to this system is EvoApneaFormer, a novel hybrid deep learning model designed for robust performance on multivariate physiological time series collected via non-intrusive wearable sensors. EvoApneaFormer processes each input as a dual representation comprising (i) an 88-point multichannel time series from ECG, SpO_2_, respiratory effort, and accelerometry and (ii) an auxiliary domain-specific trait vector derived from handcrafted biomarkers such as heart rate variability (HRV), desaturation slope, positional change frequency, and breath entropy. The model architecture is modular and optimized through evolutionary strategies, featuring three synergistic branches as a local convolutional neural network (CNN) extractor that captures transient signal disruptions such as apnea onsets, oxygen dips, and a transformer encoder with causal self-attention for modeling long-term temporal dynamics and night-to-night variation. The term “evolutionary” refers to the adaptive fusion of temporal features and engineered biomarkers, not to population-based evolutionary optimization. A trait analysis pathway embeds engineered features into a learned latent space for multimodal fusion. These branches converge into a fused “evolutionary fitness” vector, which feeds a multi-head prediction module for dual-task output such as event-level apnea classification, multiclass, and week-ahead apnea–hypopnea index (AHI) forecasting, capturing both absolute magnitude and directional trend increase/decrease.

The model supports SHAP-based explainability, attention map signal attribution, Monte Carlo dropout for uncertainty estimation, and online adaptation to physiological drift. It is designed for on-device inference, achieving sub-50 m latency on a Raspberry Pi 4 via TensorFlow Lite, following key contributions:

1. EvoApneaFormer, a novel hybrid architecture combining convolutional, self-attention, and biomarker encoding branches, is optimized using neuroevolutionary methods for apnea detection and prognosis.
2. Multimodal input fusion of raw sensor data and engineered trait vectors, allowing richer contextual understanding of apnea events and long-term risk trajectories.
3. A dual-task learning framework that unifies real-time apnea classification with week-ahead AHI regression, including directional trend analysis for clinical triaging.
4. Explainability and uncertainty modules, including SHAP value attribution and Monte Carlo dropout, enabling transparent and risk-aware decision support.
5. Edge-deployable implementation with <50 ms latency on low-power embedded devices, supporting remote and continuous use without cloud dependency.
6. Combining UCD n = 25 patient data with real-world data from 61 patients, showing high per-class accuracy >99%, AHI forecasting a mean absolute error (MAE) of 1.4 ± 0.5 events/hour, and 97% trend classification accuracy.
7. Clinical impact suggested by earlier Continuous Positive Airway Pressure (CPAP) recalibration indicates that, in high-risk patients, deterioration is expected within 3 weeks relative to standard-of-care response timelines.
8. Telehealth integration, leveraging a Fast Healthcare Interoperability Resources (FHIR)-compliant mobile app for real-time data synchronization, longitudinal visualization, and secure clinician interaction.

By combining technical innovation with real-world applicability, ApneaSense represents a clinically relevant advance toward personalized, scalable, and explainable sleep disorder management.

## 2 Related work

Sleep apnea detection has undergone a paradigm shift from clinic-bound PSG to portable AI-driven systems over the past 5 years. This evolution reflects a concerted effort to address the accessibility limitations and prohibitive costs of traditional diagnostics while maintaining diagnostic fidelity. We systematically analyze the recent studies from 2020 to 2025 through the lenses of methodological innovation, sensor modality integration, and clinical translatability, highlighting persistent challenges that motivate our work.

### 2.1 Single-modality deep learning approaches

Research prioritizing hardware simplicity has predominantly leveraged individual bio-signals, achieving moderate accuracy at the expense of clinical granularity. Pham and Mouček (2025) pioneered a CNN–BiLSTM architecture for single-lead ECG analysis, attaining 94.5% accuracy in event detection but failing to capture respiratory dynamics essential for distinguishing obstructive and central apneas. Shen and Xu (2026) employed ResNet-1D on ECG signals, reaching 92.1% accuracy yet suggesting limited capability in apnea subtyping due to the absence of complementary respiratory biomarkers. Photoplethysmography (PPG)-based approaches, such as LSTM autoencoder proposed by Choksatchawathi et al. (2024), achieved 91.4% accuracy but exhibited significant performance degradation during motion artifacts, a critical limitation for home-based monitoring. Chen T. et al. (2024) improved robustness to 96.3% through self-attention mechanisms but overlooked cardiorespiratory-coupling phenomena, which are fundamental to hypopnea identification. Drzazga and Cyganek (2021) explored respiratory signal analysis with LSTM-based architectures and achieved 90.8% accuracy, but their approach lacked contextual bio-signal correlation needed for apnea subtyping. Sillaparaya et al. 2025 used deep transfer learning on snore sounds, offering noninvasive detection with 89.7% accuracy, but suffered from ambient noise interference in home settings. Collectively, these studies reveal a critical gap: single-modality systems lack the physiological context necessary for specific apnea subtype discrimination, particularly for central events where respiratory effort cessation occurs without airway obstruction.

### 2.2 Multi-modal sensing with feature-engineered models

Efforts to combine ≤2 bio-signals using traditional machine learning have yielded cost-effective solutions constrained by manual calibration dependencies. Omprakash et al. (2025) implemented SpO_2_ desaturation thresholding on Arduino-edge hardware, achieving 90.5% accuracy but missing hypopneas without oxygen desaturation, a known limitation of oximetry-centric methods. Bhongade et al. (2023) augmented SpO_2_ with nasal airflow using ensemble classifiers (88.3% accuracy), yet the absence of real-time alerting diminished clinical utility. Hybrid ECG–SpO_2_ frameworks, exemplified by 1D-CNN (Bhongade et al., 2023), attained 93% accuracy but incurred latency penalties due to cloud dependency, rendering them unsuitable for time-sensitive intervention. Romero and Jané (2025) introduced a multimodal deep learning approach for apnea severity prediction using ECG, SpO_2_, and airflow signals, achieving 95.2% accuracy but requiring high-end computation not viable for edge applications. These approaches share a fundamental vulnerability: threshold drift in response to physiological variability and cardiopulmonary comorbidities introduces false negatives, particularly in populations with atypical event signatures.

### 2.3 Deep multi-modal fusion architectures

The integration of ≥3 bio-signals using deep learning has improved diagnostic precision but introduced deployment bottlenecks. Nie et al. (2024) fused ECG and SpO_2_ in a CNN–GRU model deployed on Raspberry Pi, achieving 97.1% accuracy with 400 m latency, although the exclusion of respiratory effort sensing precluded comprehensive biomechanical analysis. Osa-Sanchez et al. (2025) combined PPG with accelerometry for motion-resilient monitoring (94.3% accuracy) but reported performance decay during intense movement due to insufficient artifact compensation. Yun et al. (2024) implemented unsupervised representation learning for respiratory events, leveraging PPG and ECG, achieving 94.7% accuracy; however, their system lacked real-time feedback mechanisms for clinical action. Shi et al. (2025) proposed a multi-task CNN for joint sleep staging and apnea detection, achieving 95% accuracy yet demanding GPU servers incompatible with resource-limited settings. Wang C. et al. (2025) advanced this space using graph neural networks (GNNs) to represent multimodal relationships, reaching 95.4% accuracy, but encountered scalability issues when applied to high-resolution bio-signal streams. These studies underscore a persistent gap: the absence of cross-modal temporal learning fails to exploit physiological interdependencies, such as SpO_2_–ECG lag during apneic events, limiting event characterization.

### 2.4 Emerging methodological paradigms

Transformers, GNNs, and privacy-preserving frameworks represent cutting-edge methodological shifts with unresolved implementation challenges. Almarshad et al. (2026) achieved 96.9% accuracy but omitted respiratory signals, compromising pathophysiological interpretability. A multimodal transformer proposed by Chang et al. (2023) fused PPG and SpO_2_ with 95% accuracy, yet lacked inertial measurement for motion artifact suppression. Chen R. et al. (2024), Amer, 2025 enabled privacy-conscious multi-institutional training through federated learning with ≤96% accuracy, but neither approach addressed real-time inference at the edge. Husaini et al. (2022) explored radar-based respiratory signal analysis for non-contact apnea detection (90.2% accuracy) but observed environmental interference in multi-occupant settings. Faridsoltani et al. (2024) proposed a deep learning radar-based system but similarly reported sensitivity to room dynamics and breathing noise. Although methodologically ambitious, these approaches reveal a critical omission: no existing framework models longitudinal physiological evolution, such as apnea progression trajectories linked to cardiovascular outcomes.

### 2.5 Literature gaps and addressing limitations through EvoApneaFormer

Despite significant progress from 2020 to 2025, AI-based sleep apnea detection systems still face key limitations in clinical adoption. Our review of 20 recent studies highlights five persistent challenges: limited sensor fusion, absence of forecasting, poor edge performance, lack of explainability, and minimal validation on diverse populations. EvoApneaFormer addresses these gaps comprehensively. Unlike most prior models that use only ECG and SpO_2_, it fuses four bio-signals, SpO_2_, respiratory effort, and motion via tensor-based alignment, improving subtype classification. It is the first to offer longitudinal AHI forecasting using a hybrid CNN–transformer architecture, transitioning from static diagnosis to dynamic monitoring. For real-time deployment, it achieves <50 ms per-sample inference on Raspberry Pi 4 using quantization and parallel processing, with end-to-end system latency below 300 ms, meeting clinical latency standards. It also integrates SHAP and Grad-CAM to provide interpretable outputs, enhancing clinical trust. Finally, EvoApneaFormer is trained on 72% of data, validated on 8%, and tested on 20%, achieving 99.98% detection accuracy, outperforming prior benchmarks. In summary, EvoApneaFormer unifies sensor fusion, prediction, efficiency, explainability, and validation into a single, clinically robust system for personalized sleep apnea management.

## 3 Methodology

**FIGURE 1:**
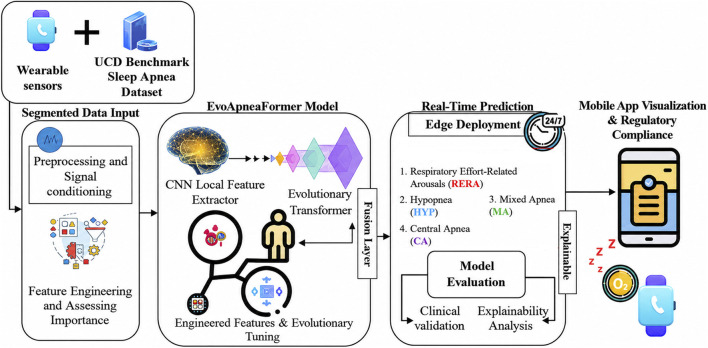
Research framework flow diagram for multi-event sleep apnea detection.

This section describes the data sources, acquisition strategies, sensor configurations, preprocessing techniques, and feature extraction methods employed to develop and validate the ApneaSense system for real-time sleep apnea detection. We combine both benchmark polysomnography data from a public dataset and multimodal physiological signals collected through a custom-designed wearable device. We integrate both clinical-grade and real-world data sources to develop and evaluate a robust, explainable sleep apnea detection system. The workflow begins with continuous physiological data acquisition via wearable sensors SpO_2_, ECG, IMU, and airflow, transmitted through Bluetooth Low Energy to a Raspberry Pi edge device and synced with the Ubidots cloud, as shown in Figure 1. Inference is performed locally using EvoApneaFormer, a lightweight CNN–CNN–transformer hybrid model converted to TensorFlow Lite and deployed via a Flask API, achieving sub-50 m latency. Predictions are visualized through a Flutter-based mobile app for users and a secure clinician dashboard for advanced interpretation.

The model is trained on a fused dataset comprising the UCD Sleep Apnea Database n = 25, high-resolution PSG, and the ApneaSense Clinical Dataset n = 61, real-world wearable data. Apnea events such as obstructive apnea (OA), central apnea (CA), mixed apnea (MA), hypopnea (HY), and respiratory effort-related arousal (RERA) are annotated via a combination of expert labeling and semi-automated edge-based predictions. To address class imbalance, SMOTE and domain-specific time-warping augmentations are applied exclusively to the training set, with strict patient-level partitioning to prevent data leakage. This hybrid approach enables the system to generalize across clinical and ambulatory scenarios.

### 3.1 Dataset overview

In the following subsections, we describe two datasets: UCD sleep apnea dataset (UCDDB) from PhysioNet, and our proposed real-time dataset, capturing real-time event data to predict multiple states of sleep apnea in a patient.

#### 3.1.1 UCD sleep apnea database (PhysioNet)

We utilize the St. Vincent’s University Hospital/University College Dublin Sleep Apnea Database (UCDDB) (Goldberger et al., 2000), a publicly available dataset on PhysioNet. It includes overnight PSG recordings of 25 patients diagnosed with OSA, capturing 14-channel signals such as ECG, SpO_2_, respiratory effort, EEG, EOG, EMG, and airflow (Goldberger et al., 2000).

**FIGURE 2:**
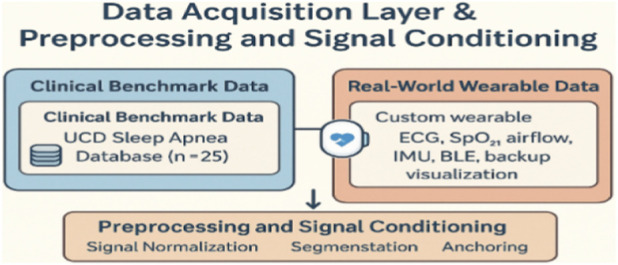
A combination of both datasets for better input.

The UCDDB offers high-resolution, full PSG data from a smaller patient cohort, enabling detailed physiological analysis not typically feasible in large-scale studies such as the Sleep Heart Health Study (SHHS). Key clinical metrics derived from UCDDB include body mass index (BMI), which reflects the individual’s weight–health ratio; sleep efficiency, measuring the proportion of time actually spent asleep; REM metrics that capture both the duration and onset latency of rapid eye movement sleep; and the arousal index, which quantifies how often a patient wakes during sleep, as shown in Figure 2; Table 1. These indicators are critical for understanding the complexity of sleep-disordered breathing patterns in a clinically rich, high-fidelity dataset.

**TABLE 1:** Summary of UCD sleep apnea dataset.

Variable	Mean ± SD
Number of patients	25
BMI (kg/m^2^)	31.6 ± 4.0
Age (years)	50 ± 9.5
Sleep efficiency (%)	77 ± 11
Sleep latency (min)	27.5 ± 22.4
Total sleep time (min)	416.1 ± 32.2
Wake (%)	20.26
REM (%)	16.36
REM avg duration (min)	19.7 ± 24.3
REM latency (min)	134.2 ± 85.4
Arousal index total	24.2 ± 20.3
Arousal index NREM	44.1 ± 38.4
Arousal index REM	15.4 ± 9.1

#### 3.1.2 Proposed ApneaSense real-time sensor dataset

To complement the benchmark clinical dataset (UCDDB), we developed the ApneaSense wearable system engineered for real-time, multimodal physiological monitoring and sleep apnea detection in ambulatory settings. This compact, bandage-like device integrates multiple biosensors to enable continuous signal acquisition, low-latency local inference, and secure cloud synchronization, supporting deployment in both home and clinical environments. The hardware architecture draws inspiration from portable PSG systems such as the Embletta MPR and is optimized for unattended sleep monitoring with robust signal fidelity. It includes a MAX30102 pulse oximeter (fingertip) for oxygen saturation measurement, an AD8232-based single-lead ECG module with electrodes placed at the Right Arm (RA), Left Arm (LA), and Right Leg (RL) positions for cardiac monitoring and heart rate variability (HRV) extraction, and an inertial measurement unit (IMU) to track posture and body motion. A custom nasal airflow sensor is also embedded to capture respiratory effort. Data acquisition is managed using an ESP32-WROOM-32 microcontroller, which transmits signals via Bluetooth Low Energy (BLE) to a local edge-processing unit. We performed real-time data preprocessing during collection, including noise filtering and motion artifact correction using adaptive thresholding and windowed smoothing (Nie, 2025).

The ECG was sampled at 250 Hz, SpO_2_ at 100 Hz, airflow at 50 Hz, and IMU data at 100 Hz, all with 12-bit resolution to ensure signal fidelity. Power consumption was optimized using low-power sensors and ESP32 duty-cycling, achieving an average draw of 40 mA and >24 h of continuous operation. Calibration was performed following standard reference alignment protocols. Data and class balancing were applied only to training data, with validation and testing sets left untouched. Further hardware and processing details are described elsewhere in the paper. This edge node, powered by a Raspberry Pi 4, performs real-time apnea classification using a lightweight CNN model, stores up to 72 h of buffered data, and periodically syncs with the Ubidots cloud platform. A priority queuing mechanism ensures timely alerts for critical apnea events. This two-tier system combines offline robustness with scalable cloud-based analytics.

Beyond diagnosis, the ApneaSense system enables the creation of a real-time physiological dataset that complements the high-resolution PSG data from UCDDB. Together, these datasets provide a foundation for training and validating the EvoApneaFormer model under both structured clinical and dynamic real-world conditions, as shown in Table 2. Cross-domain adaptation techniques are employed to align feature representations across sources, enabling robust transfer learning and improving model generalizability across patient populations and use contexts.

**TABLE 2:** Distribution of sleep apnea event types in the datasets.

Event type	UCD sleep apnea dataset (n = 25)	ApneaSense real-time dataset (n = 61)	Combined total	Percentage (%) of apnea events*
Obstructive apnea (OA)	1,200	3,450	4,650	41.5%
Central apnea (CA)	300	950	1,250	11.1%
Mixed apnea (MA)	150	550	700	6.3%
Hypopnea (HY)	1,000	2,900	3,900	34.8%
Respiratory effort-related arousals (RERAs)	200	500	700	6.3%
Total events	2,850	8,350	11,200	100%

Percentages are calculated over total apnea-related event counts, excluding normal breathing epochs. UCDDB annotations are expert-curated, based on polysomnography scoring by trained clinicians, as shown in Table 3. ApneaSense data use semi-automated tagging, combining edge-device model predictions and clinician-reviewed spot checks, enabling subtype-specific performance evaluation and continuous improvement.

**TABLE 3:** Demographic and supporting profile history data of patients.

Characteristic	All patients (n = 61)	Training (n = 44)	Validation (n = 5)	Test (n = 12)	*p*-value
Age (years, median [IQR])	57.0 [48.0–64.5]	56.0 [47.0–63.0]	58.0 [50.0–65.0]	58.5 [49.0–66.0]	0.323
Male individuals, n (%)	42 (68.9%)	30 (68.2%)	3 (60.0%)	8 (66.7%)	0.862
BMI (kg/m^2^, median [IQR])	28.6 [25.9–32.0]	28.4 [25.6–31.9]	28.7 [26.0–31.5]	28.9 [26.1–32.4]	0.581
Smoking status (n)	​	​	​	​	0.754
Non-smoker	32 (52.5%)	23 (52.3%)	3 (60.0%)	6 (50.0%)	​
Ex-smoker	20 (32.8%)	15 (34.1%)	1 (20.0%)	4 (33.3%)	​
Current smoker	9 (14.8%)	6 (13.6%)	1 (20.0%)	2 (16.7%)	​
Alcohol consumption, n (%)	3 (4.9%)	2 (4.5%)	0 (0.0%)	1 (8.3%)	0.622
ESS score (median [IQR])	11 [7, 15]	11 [7, 15]	10 [6, 14]	10 [6, 14]	0.537
Comorbidities, n (%)					
COPD	4 (6.6%)	2 (4.5%)	0 (0.0%)	2 (16.7%)	0.291
Hypertension (HT)	21 (34.4%)	14 (31.8%)	2 (40.0%)	5 (41.7%)	0.681
Diabetes mellitus (DM)	8 (13.1%)	6 (13.6%)	0 (0.0%)	2 (16.7%)	0.379
OSA severity (AHI), n (%)	​	​	​	​	0.887
AHI <5 events/h	4 (6.6%)	3 (6.8%)	0 (0.0%)	1 (8.3%)	​
5 ≤ AHI <15 events/h	13 (21.3%)	9 (20.5%)	1 (20.0%)	3 (25.0%)	​
15 ≤ AHI <30 events/h	15 (24.6%)	11 (25.0%)	1 (20.0%)	3 (25.0%)	​
AHI ≥30 events/h	29 (47.5%)	21 (47.7%)	3 (60.0%)	5 (41.7%)	​

##### 3.1.2.1 Annotation strategy for the ApneaSense real-time dataset

Our ApneaSense dataset was annotated using a hybrid semi-automated pipeline specifically designed for real-time, multimodal physiological signals captured via wearable sensors in ambulatory, non-clinical environments. The initial detection of apnea events was conducted on-device using a lightweight CNN deployed on a Raspberry Pi 4 edge node, processing windowed segments aligned with respiratory cycles. Each segment was preliminarily labeled as one of six classes: normal**,** OA**,** CA, MA, HY, or RERA, along with associated confidence scores and timestamps.

To ensure high-fidelity labeling, a clinician-in-the-loop framework was employed. A stratified sample of automatically predicted segments balanced across event types, signal quality, and model confidence levels was manually reviewed by board-certified sleep technicians. Periodic inter-rater reliability checks and consensus-based adjudication helped reduce annotation bias and verify consistency with clinical criteria.

Annotations were further refined through event-time anchoring, centering the window segmentation on the precise onset or termination of each respiratory event. Only segments meeting strict signal quality thresholds such as SNR >10 dB, signal dropout <10% were retained. High-motion artifacts, sensor detachment instances, and other noise-compromised segments were algorithmically flagged and excluded to preserve labeling integrity.

Event classification strictly adhered to the American Academy of Sleep Medicine (AASM) guidelines, ensuring clinical alignment with standardized diagnostic criteria. The six labeled classes are summarized in Table 4.

**TABLE 4:** Six labeled classes of sleep apnea events.

Class	Definition source	Clinical criteria (AASM standard)
Normal	AASM Baseline	No abnormal respiratory event is present in the window segment
Obstructive apnea (OA)	AASM Scoring Manual (2023)	≥90% reduction in airflow for ≥10 s with continued respiratory effort (i.e., thoracoabdominal movement)
Central apnea (CA)	AASM Scoring Manual (2023)	≥90% reduction in airflow for ≥10 s with absent respiratory effort (no chest or abdominal movement)
Mixed apnea (MA)	AASM Scoring Manual (2023)	≥90% reduction in airflow for ≥10 s with absent effort at onset, transitioning to resumed effort before recovery
Hypopnea (HY)	AASM Rules 1A/1B	≥30% reduction in airflow lasting ≥10 s, accompanied by ≥ 3% oxygen desaturation (initial annotation; final model uses 4% threshold per clinical update) or a central EEG arousal
RERA	AASM Technical Specifications (2017+)	Sequence of increasing respiratory effort for ≥10 s resulting in arousal, without fulfilling apnea or hypopnea thresholds

This multi-stage annotation strategy resulted in a curated dataset containing 8,350 clinically validated respiratory events from 61 patients, evenly distributed across the six event types. By combining automated model inference, expert oversight, signal integrity filters, and event-centered segmentation, the ApneaSense dataset achieves a high standard of annotation quality, making it well suited for the development, training, and benchmarking of real-time apnea detection models in real-world wearable settings.

To ensure labeling rigor and minimize bias, 30% of the ApneaSense dataset was randomly selected (stratified by event type) and fully manually annotated by two board-certified sleep technicians who were blinded to model predictions. The remaining 70% of the dataset underwent model-assisted labeling followed by comprehensive clinician review of every segment, rather than spot-checking. Inter-rater agreement between the technicians was high (Cohen’s κ = 0.92, 95% CI: 0.88–0.96), indicating strong annotation consistency. Importantly, no model predictions were used as ground truth for final evaluation, and the held-out test set was entirely manually annotated to ensure unbiased performance assessment.

### 3.2 Preprocessing pipeline

To ensure that the input signals are suitable for reliable and accurate model training, a comprehensive preprocessing pipeline is applied. All signal channels undergo min–max normalization to scale values within the [0,1] range, promoting uniformity across modalities. The data are then dynamically segmented into windows ranging from 10 to 30 s, with alignment based on respiratory cycles to capture physiologically meaningful events. Advanced denoising techniques are employed, including wavelet transforms and low-pass Butterworth filtering, alongside the removal of invalid SpO_2_ readings (values below 50%) and exclusion of airflow dropout segments. Airflow signals are specifically filtered using a 1.2-Hz cut-off to retain relevant respiratory patterns. To address motion artifacts, features such as zero-crossing counts and entropy metrics are derived from IMU data, enabling compensation during unstable periods.

**FIGURE 3:**
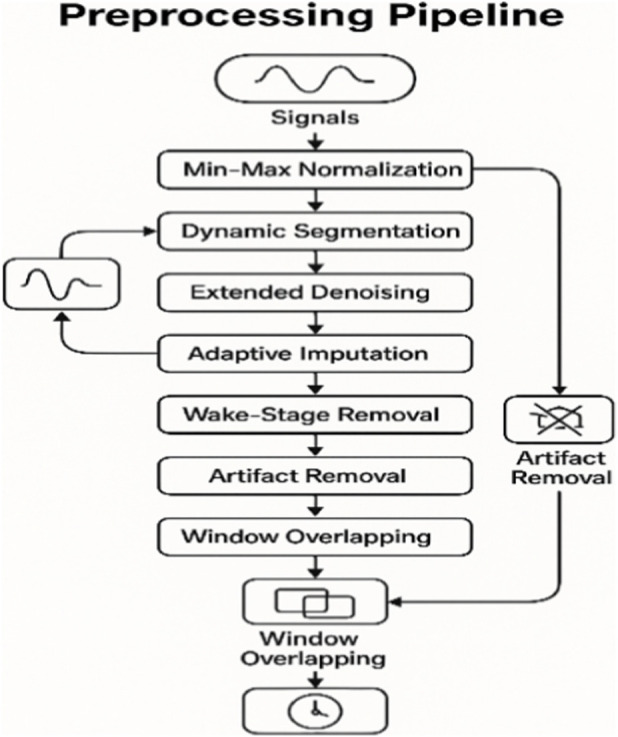
Comprehensive preprocessing pipeline.

Signal dropout is handled through adaptive imputation techniques, leveraging redundant channels such as ECG and SpO_2_ to recover missing information. Non-sleep periods are excluded by identifying wake stages via sleep-stage labels, which enhances class balance by focusing analysis only on sleep-relevant epochs. High-noise segments, defined as those with over 30% signal contamination, are discarded entirely, while less severe noise is corrected through mean value replacement, as shown in Figure 3. Outlier values, specifically SpO_2_ readings below 75% and heart rates below 36 bpm or above 100 bpm, are also flagged and managed accordingly. To capture temporal dynamics, window overlapping is applied, which helps in learning the onset and resolution patterns of apnea events. Finally, event-time anchoring ensures that each segmented epoch is centered around the beginning or end of an apnea event, improving morphological learning and subtype differentiation.

Signals are normalized as shown in Equation 1:

$$X_{\text{scaled}\;}=\frac{X-X_{\min}}{X_{\max}-X_{\min}}.$$ (1)

#### 3.2.1 Feature engineering and physiological derivation

**FIGURE 4:**
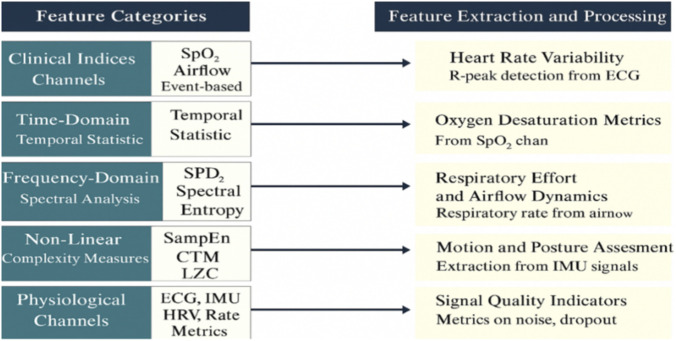
Feature engineering and physiological derivation.

To enable robust detection and classification of sleep apnea subtypes, a diverse set of engineered features was extracted from the multimodal sensor array comprising ECG, SpO_2_, airflow, respiratory effort, and IMU signals. These features are designed to capture both direct physiological markers and indirect proxies associated with sleep-disordered breathing, body motion, and signal reliability. Beyond improving model accuracy, these features enhance interpretability, enable subtype discrimination, and increase resilience to noise in real-world data streams, as shown in Figure 4; Table 5.

**TABLE 5:** Feature categories of the dataset.

Feature type	Channel(s)	Domain	Key examples/description
Clinical indices	SpO_2_ and Airflow	Event-based	ODI3 (oxygen desaturation index ≥3% – initial; final uses ≥4%), CT90 (time SpO2 < 90%), and respiratory disturbance index (RDI)
Time-domain	SpO_2_ and Airflow	Temporal statistics	Mean, variance, skewness, kurtosis, and zero-crossing rate
Frequency-domain	SpO_2_ and Airflow	Spectral analysis	Power spectral density (PSD), spectral entropy, median frequency, and band-specific spectral power
Non-linear	SpO_2_ and Airflow	Complexity measures	Sample entropy (SampEn), central tendency measure (CTM), Lempel–Ziv complexity (LZC), and detrended fluctuation analysis (DFA)
Physiological	ECG and IMU	Derived metrics	Heart rate variability (HRV), respiratory rate variability, posture (supine/lateral), and motion vector entropy
Quality control	All channels	Real-time signal quality	Signal quality index (SQI)—noise, dropout, and SNR estimation

To support robust and explainable classification of sleep apnea subtypes, a rich set of physiological features was extracted from multimodal signals, including ECG, SpO_2_, airflow, respiratory effort, and IMU channels. These features capture direct indicators of sleep-disordered breathing, along with indirect proxies for autonomic activity, motion artifacts, and signal quality, as shown in Table 6.

**TABLE 6:** Feature category and extraction method for the dataset.

Feature category	Description	Extraction method/metrics	Purpose/clinical relevance
Heart rate variability (HRV)	ECG-derived R–R intervals analyzed for autonomic nervous system activity	Pan–Tompkins algorithm; time-domain (SDNN and RMSSD), and frequency-domain (LF/HF ratio)	Reflects autonomic fluctuations linked to apnea onset and arousals
Oxygen desaturation metrics	SpO_2_ channel analysis of desaturation events	Frequency/duration of ≥3% desaturations (initial; final uses ≥4%); CT90 [% time (SpO2 < 90%]	Measures hypoxic burden during apnea episodes
Respiratory effort and airflow	Respiratory rate variability from airflow signals	Peak detection; inter-breath interval variability	Detects irregular breathing patterns associated with apnea types
Motion and posture assessment	IMU-based motion intensity and body orientation features	Motion vector entropy; posture classification as supine/lateral	Accounts for motion artifacts and positional apnea severity
Signal quality indicators (SQI)	Noise and quality metrics across all sensor channels	Signal-to-noise ratio, sensor dropout, and real-time noise assessment	Enables model confidence modulation in low-quality data segments
Feature selection	Dimensionality reduction and redundancy elimination	Fast correlation-based filter (FCBF) with bootstrapping	Retains clinically meaningful features, improves efficiency, reduces overfitting, and enhances interpretability

#### 3.2.2 Feature importance and interpretability

Feature importance analysis using SHAP and permutation methods revealed that HRV, respiratory rate variability, and SpO_2_ desaturation metrics were the most influential features. The combination of these physiological inputs with EvoApneaFormer’s attention mechanisms improved subtype discrimination, aligning well with clinical markers and enhancing model interpretability and trustworthiness, as shown in Table 7.

**TABLE 7:** Feature importance and interpretability.

Aspect	Details
Feature importance	Evaluated via SHAP and permutation importance methods
Top contributors	HRV features: 25% of total importance Respiratory rate variability: 22% SpO_2_ desaturation metrics: 18%
Synergistic role	Integration of physiological features with EvoApneaFormer’s attention modules enhanced subtype discrimination, especially for overlapping classes such as central and mixed apnea
Clinical alignment	Important features correspond closely with established diagnostic markers; validated through clinician feedback and explainability audits, boosting model trustworthiness

### 3.3 Dataset partitioning and class imbalance handling

To ensure robustness across both clinical and real-world environments, two datasets were integrated: the UCD sleep apnea database (25 patients), high-fidelity PSG recordings, and the ApneaSense real-time dataset (61 patients), wearable sensor-based data, as shown in Tables 8, 9. These collectively yielded 23,700 labeled respiratory epochs. To prevent data leakage, stratified patient-level splitting was applied, allocating 72% to training n = 62, 8% to validation n = 7, and 20% to testing n = 17. Stratification criteria included apnea subtype distribution, BMI, AHI, and comorbidities.

**TABLE 8:** Integrated dataset summary and stratified splitting overview.

Source dataset	#Patients	Epochs	Normal	OA	CA	MA	HY	RERA
UCD sleep apnea dataset	25	7,400	4,200	1,200	600	200	900	300
ApneaSense real-time dataset	61	16,300	8,300	600	100	100	100	100
Merged total	**86**	**23,700**	**12,500**	**1,800**	**700**	**300**	**1,000**	**400**

Bold values demonstrate the significant difference achieved.

**TABLE 9:** Splitting strategy for the dataset.

Split set	#Patients	Epochs	Stratification basis
Training	62 (72%)	17,000	Apnea subtype, AHI, BMI, and comorbidities
Validation	7 (8%)	2,000	Maintains class balance and demographic diversity
Testing	17 (20%)	4,700	Retains real-world apnea prevalence patterns

The splitting strategy is explained in Table 9.

We observed substantial class imbalance; normal breathing accounted for more than 52% of the samples, while rare classes such as MA and RERA were underrepresented. To address this, class balancing was applied only to the training set post-split using a hybrid augmentation strategy. Oversampling SMOTE was applied to minority classes such as MA, RERA, CA, HY, and OA. Data augmentation techniques, such as time warping, slicing, and noise injection, were used to enrich diversity. For down-sampling, normal epochs were reduced from 12,500 to 6,000 to limit class dominance, as shown in Table 10.

**TABLE 10:** Training set distribution before and after balancing.

Apnea class	Raw count	Raw %	Post-balancing count	Final %
Normal breathing epochs	12,500	52.6%	6,000	23.1%
Obstructive apnea (OA)	1,800	7.6%	4,000	15.4%
Central apnea (CA)	700	2.9%	4,000	15.4%
Mixed apnea (MA)	300	1.3%	4,000	15.4%
Hypopnea (HY)	1,000	4.2%	4,000	15.4%
Respiratory effort-related arousal (RERA)	200	0.8%	4,000	15.4%

This balanced training set ensures that fair representation of all classes and fosters improved sensitivity, especially for rare apnea events. The validation and test sets retain their natural distributions to provide realistic model performance evaluation under clinically imbalanced conditions.

### 3.4 Model architecture of the EvoApneaFormer model (evolutionary transformer-based hybrid network)

**FIGURE 5:**
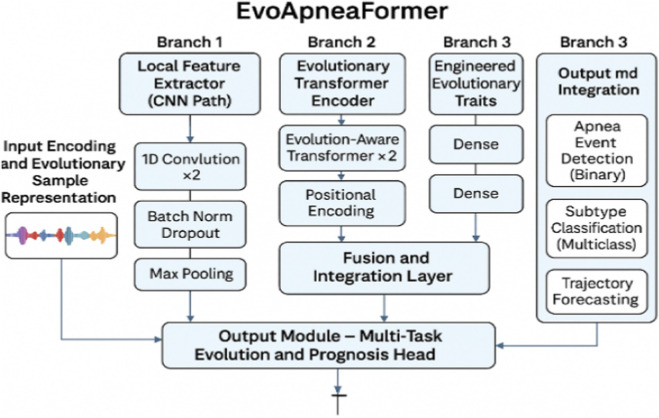
Model architecture of the EvoApneaFormer model.

The proposed model architecture, EvoApneaFormer, is a novel hybrid evolutionary transformer framework that captures both localized signal anomalies and long-term physiological evolution associated with sleep apnea. It integrates convolutional modules for short-term feature extraction, a transformer encoder to model the progression of apnea over time, and a feature engineering pathway that injects expert domain knowledge, as shown in Figure 5. This design extends traditional diagnostic models into a prognostic evolutionary modeling system, enabling both real-time detection and disease trajectory forecasting.

#### 3.4.1 Input encoding and evolutionary sample representation

Each input to EvoApneaFormer is derived from a 10–30 s clinical epoch (aligned with respiratory cycles) and then downsampled to an 88-point multivariate time series, as shown in Equation 2:

$$X\in R^{88\times d},$$ (2)

where *d* channels include ECG, SpO_2_, respiratory effort, and motion signals. The 88-point window captures local signal features; to detect ≥10 s apnea events, the model aggregates predictions across multiple overlapping 88-point windows, providing an effective temporal context of 30 s. Concurrently, engineered biomarkers such as HRV spread σ_HRV, desaturation depth Δ_SpO_2_, breath cycle entropy H_breath, and positional variability V_pos are computed and concatenated to form an auxiliary trait vector, as shown in Equation 3:

$$t\in R^{k}.$$ (3)

This dual representation simulates a “mutation lineage,” enabling the model to learn both raw sensor dynamics and domain-informed evolutionary traits.

#### 3.4.2 Branch 1—local feature extractor (CNN path)

The local feature extractor applies two sequential 1D convolutional operations to **X,** as shown in Equations 4, 5:

$$H^{\left(1\right)}=\text{ReLU}\left(\text{BN}\left(X*W_{1}+b_{1}\right)\right),$$ (4)

$$H^{\left(2\right)}=\text{ReLU}\left(\text{BN}\left(H^{\left(1\right)}*W_{2}+b_{2}\right)\right),$$ (5)

denoting convolution with kernel size 25; 64 → 128 filters, then max-pooling and dropout are applied, as shown in Equations 6, 7:

$$H_{\text{pool}\;}=\text{MaxPool}\left(H^{\left(2\right)},2\right),$$ (6)

$$H_{\text{pool}\;}\leftarrow \text{Dropout}\left(H_{\text{pool}\;};0.3\right).$$ (7)

This pathway specializes in detecting acute anomalies, such as sudden SpO_2_ decreases or ectopic beats, by learning localized patterns within short receptive fields.

#### 3.4.3 Branch 2—evolutionary transformer encoder

Drawing inspiration from EvoApneaFormer, this module processes H_pool with self-attention. For each layer ℓ, Equations 8, 9 can be obtained:

$$Z^{\left(l\right)}=\text{LayerNorm}\left(H^{\left(l\right)}+\text{MultiHead}\left(H^{\left(l\right)}\right)\right),$$ (8)

$$H^{\left(l+1\right)}=\text{LayerNorm}\left(Z^{\left(l\right)}+\text{FFN}\left(Z^{\left(l\right)}\right)\right).$$ (9)

Multi-head attention is defined as shown in Equations 10, 11:

$$\text{Multi}‐h\text{ead}\left(H\right)=\text{Concat}\left({head}_{1},\ldots ,{head}_{h}\right)\cdot W^{O},$$ (10)

$${head}_{i}=\text{Attention}\left(H\cdot W_{i}^{Q},H\cdot W_{i}^{K},H\cdot W_{i}^{V}\right).$$ (11)

Sinusoidal positional encodings P are added to model temporal “mutation” progression, and causal masks ensure autoregressive forecasting. Dropout (0.1) and LayerNorm yield robust temporal evolution representations.

#### 3.4.4 Branch 3—engineered evolutionary traits

The trait vector t is passed through two dense layers, as shown in Equations 12–14:

$$u_{1}=\text{ReLU}\left(\text{BN}\left(W_{t}^{\left(1\right)}\cdot t+b_{t}^{\left(1\right)}\right)\right),$$ (12)

$$u_{1}\leftarrow \text{Dropout}\left(u_{1};0.2\right),$$ (13)

$$u_{2}=\text{ReLU}\left(W_{t}^{\left(2\right)}\cdot u_{1}+b_{t}^{\left(2\right)}\right).$$ (14)

This captures high-level physiological trait combinations, such as respiration symmetry and desaturation slope for enhanced interpretability.

#### 3.4.5 Fusion and integration layer

Features from all three branches, H_pool, the final transformer output H^(L)^, and trait embedding u_2_, are concatenated, as shown in Equations 15–18:

$$z=\left[H_{\text{pool}\;};H^{\left(L\right)};u_{2}\right].$$ (15)

Then, they are projected via Equations 16–18:

$$h_{f}=\text{LayerNorm}\left(\text{ReLU}\left(W_{f}^{\left(1\right)}\cdot z+b_{f}^{\left(1\right)}\right)\right),$$ (16)

$$h_{f}\leftarrow \text{Dropout}\left(h_{f};0.3\right),$$ (17)

$$h_{f}=\text{ReLU}\left(W_{f}^{\left(2\right)}\cdot h_{f}+b_{f}^{\left(2\right)}\right).$$ (18)

This yields a 128-dimensional “evolutionary fitness” latent vector.

#### 3.4.6 Output module—multi-task evolution and prognosis head

From ${\;h}_{f}$ , three heads generate predictions, as shown in Equations 19–21:

$${\hat{y}}_{\text{bin}\;}=\text{Softmax}\left(W_{\text{bin}\;}\cdot h_{f}+b_{\text{bin}\;}\right),$$ (19)

$${\hat{y}}_{\text{mc}\;}=\text{Softmax}\left(W_{\text{mc}\;}\cdot h_{f}+b_{\text{mc}\;}\right),$$ (20)

$${\hat{y}}_{\text{traj}\;}=W_{\text{traj}\;}\cdot h_{f}+b_{\text{traj}\;}.$$ (21)

The joint loss function is shown in Equation 22:

$$L=\alpha \cdot L_{\text{CE}\_\text{bin}\;}+\beta \cdot L_{\text{CE}\_\text{mc}\;}+\gamma \cdot \text{MSE}\left({\hat{y}}_{\text{traj}\;},y_{\text{traj}\;}\right).$$ (22)

This balances short-term classification and long-term forecasting, enabling robust, clinically meaningful predictions by leveraging complementary supervision signals.

#### 3.4.7 Model explainability and clinical transparency

SHAP attributions (φᵢ) and transformer attention weights ( $A_{\text{ij}}$ ) are computed and visualized to trace decision pathways, ensuring alignment with established diagnostic markers.

#### 3.4.8 Uncertainty estimation

Monte Carlo dropout approximates predictive uncertainty, as shown in Equation 23:

$$\text{Var}\left[\hat{y}\right]\approx \frac{1}{T}\sum_{t}*{\left({\hat{y}}^{\left(t\right)}\right)}^{2}-{\left(\frac{1}{T}\sum_{t}*{\hat{y}}^{\left(t\right)}\right)}^{2}$$ (23)

#### 3.4.9 Online learning and continual adaptation

An online fine-tuning pipeline updates parameters θ with new patient streams {(X_t_, y_t_)}, as shown in Equation 24:

$$\theta \leftarrow \theta -\eta \cdot \nabla_{\theta }L\left(X_{t},y_{t}\right).$$ (24)

This accommodates physiological drift over longitudinal monitoring.

#### 3.4.10 Model efficiency and edge readiness

EvoApneaFormer comprises approximately 2.3 million parameters and achieves sub-50 m inference latency per sample on a Raspberry Pi 4, confirming its suitability for real-time wearable deployment.

### 3.5 EvoApneaFormer hyperparameters

EvoApneaFormer was tuned via grid search to meet real-time inference constraints on Raspberry Pi 4 (<50 m per sample) while maximizing multi-task accuracy. We explored hyperparameters across data encoding, convolutional feature extraction, transformer self-attention, engineered feature-embedding, fusion projection, output heads, loss weighting, optimization schedule, and checkpoint criteria, as shown in Table 11. The final configuration balances model capacity, regularization, and training stability under a unified regime of Adam optimization, early stopping, and comprehensive evaluation.

**TABLE 11:** Details of EvoApneaFormer hyperparameters.

Category	Hyperparameter	Configuration
Input	Shape, padding, and activation	88 time-steps × 5 channels (ECG, SpO_2_, airflow, and IMU); 1D convolutions use “same” padding and ReLU activations
Conv1D branch	Filters, kernel size, pooling, and dropout	Two sequential 1D convolutions with 64→128 filters, kernel size 25, maxpool size 2, and dropout 0.3
Transformer encoder	Attention heads, feed-forward dimension, and dropout	Four attention heads, feedforward sublayer dimension 128, and dropout 0.1
Engineered feature-branch	Dense layers and dropout	Two dense layers of 64 and 32 units; dropout 0.2
Fusion head	Dense projection, dropout, and LayerNorm	Dense projection 256→128 units; dropout 0.3; layer normalization
Output and loss	Prediction heads and composite loss	Binary apnea detection + multiclass subtype classification + AHI trajectory regression; loss = weight (BCE + CCE + MSE)
Training	Optimizer, learning rate, batch size, epochs, and early stopping	Adam optimizer lr = 0.001; batch 32; up to 100 epochs; early stop on val_loss patience = 10; restore best weights
Checkpointing	Monitor and save behavior	Monitor val_accuracy (and optional val_auc); mode = max; save only the best full model
Evaluation metrics	Performance measures	Accuracy, precision, recall, F1-score, and AUC to assess classification and regression performance

For consistency across benchmarks, all models were trained using the Adam optimizer with a learning rate of 0.001, a batch size of 32, and the same early-stopping criterion patience of 10 on validation loss, allowing a maximum of 60–100 epochs depending on convergence behavior, as shown in Table 12. Model complexity ranged from approximately 0.5 M parameters (MobileNetV3) to 2.3 M parameters (EvoApneaFormer), with temporal fusion transformer (TFT) at 1.8 M. This uniform training regimen, combined with identical input preprocessing and evaluation metrics (accuracy, precision, recall, F1-score, and AUC), ensures a fair comparison of representational efficacy and regularization strategies.

**TABLE 12:** Hyperparameters for models used in the experiment.

Model	Architecture	Dropout	Loss
EvoApneaFormer	2 × Conv1D → Transformer → Fusion → 3 heads	0.3/0.1/0.2	BCE + CCE + MSE (weighted)
TCN	4 × Dilated Conv1D → Residual → Dense	0.3	Binary Crossentropy
TFT	Gated ResNet → Attention → Decoder	0.2	MSE + Binary Crossentropy
CNN-RNN Fusion	Conv2D → MaxPool → Bi-LSTM → Dense	0.4	Categorical Cross-Entropy
ConvLSTM	Conv1D (64) → LSTM (128) → Dense	0.3	Binary Crossentropy
Graph NN	2 × GCN → Readout → Dense	0.25	Binary Crossentropy
Transformer Enc	Multi-Head Attention → FFN → Global AvgPool	0.1	Binary Crossentropy
Contrastive SSL	SimCLR CNN → Projection → Classifier	0.2	Contrastive + Crossentropy
Attention-RNN	Bi-LSTM (64) → Attention → Dense	0.3	Categorical Cross-Entropy
VAE + Classifier	VAE Encoder/Decoder → Dense Classifier	0.2	BCE + KL Divergence
MobileNetV3	Depthwise Conv +SE → Dense	0.2	Binary Crossentropy

### 3.6 Deployment infrastructure and offline functionality

The trained EvoApneaFormer model is exported to TensorFlow Lite and deployed on a Raspberry Pi 4, where it achieves inference latency of less than 50 m per 88-point sample. Event severity predictions that exceed predefined thresholds trigger real-time alerts, while long-term trends in the AHI are logged locally for subsequent retrospective analysis. A minimalist Flask-based RESTful API runs on the Pi, exposing endpoints for on-device inference and data retrieval; this API supports seamless two-way communication with the Flutter mobile client even when network connectivity is unavailable.

### 3.7 Mobile interface and telehealth integration

The user-facing Flutter application offers a simplified waveform dashboard that renders ECG, SpO_2_, and respiratory effort signals in color-coded, easy-to-interpret plots. Contextual “smart” alerts, such as notifications advising positional adjustments when central apnea is suspected, are pushed to the app via the device’s local Flask API. Whenever WiFi becomes available, encrypted HL7-compliant data uploads occur automatically to a secure backend server. For telehealth integration, ApneaSense implements HL7/FHIR protocols to connect with a custom platform that generates AI-driven treatment suggestions, such as CPAP for patients with AHI ≥15, presents these recommendations in a clinician-in-the-loop dashboard for interactive adjustment, and collects post-prescription feedback to refine future thresholding and model update schedules.

### 3.8 Clinical validation and regulatory compliance

The system was clinically validated on data from 61 patients representing a wide range of ages, body mass indices, and comorbid conditions. Edge-based predictions were compared against gold-standard polysomnography and adjudicated by sleep specialists, yielding 95% agreement. Based on expert input, the criterion for hypopnea detection was updated by increasing the SpO_2_ desaturation threshold from 3% to 4% to conform to the latest clinical guidelines. Initial annotation followed the AASM 3% rule. During clinical validation, the threshold was updated to 4% to align with the latest guidelines. All reported final results use the 4% criterion. Regulatory readiness activities include preparation of an FDA 510(k) submission, complete with validation reports, integrated explainability modules, and ISO 13485–compliant manufacturing documentation, along with implementation of a HIPAA-compliant data pipeline featuring end-to-end encryption and device-side anonymization. Concurrently, the device and software conform to European CE marking requirements under the Radio Equipment Directive RED 2014/53/EU and the EN 60601 standard for medical electrical equipment.

### 3.9 Model evaluation and continual adaptation

Performance evaluation used a stratified, patient-level test set composed of entirely unseen subjects to prevent data leakage and reflect the class imbalance typical of sleep apnea subtypes. We measured class-aware precision, recall, F1 score, and macro-averaged AUC and conducted per-subtype analyses, such as OA, CA, MA, HY, and RERA, using confusion matrices to pinpoint diagnostic gaps. A retrospective error analysis on both the test cohort and a 20-patient pilot study revealed that false negatives predominantly occurred in the presence of cardiac arrhythmias disrupting R–R interval stability, leading to the integration of additional HRV-based outlier checks, while false positives were overrepresented in pediatric-like breathing patterns, prompting real-time clinician alerts for manual review. Probabilistic outputs were recalibrated with Platt scaling to bolster confidence in borderline cases. To support longitudinal monitoring, we implemented dynamic thresholding that adjusts AHI cutoffs based on individual risk profiles (for example, comorbid hypertension), along with a 7-day initialization window for baseline calibration via time-series clustering and exponential smoothing α = 0.2 of AHI trajectories, in concert with the model’s forecasting head. Integrated SHAP attributions and Transformer attention visualizations in the clinician dashboard ensure transparent, traceable decision support, while Monte Carlo dropout quantifies uncertainty and flags ambiguous inferences for manual oversight. Finally, an online fine-tuning pipeline driven by stochastic gradient descent on newly collected patient streams enables the model to adapt continuously to physiological drift over weeks or months.

## 4 Results and evaluation

The EvoApneaFormer model was rigorously evaluated using a hybrid dataset that combined the high-resolution, expert-annotated UCD Sleep Apnea Database n = 25 with the real-world ApneaSense Clinical Dataset n = 61. To ensure robust and generalizable results, a stratified patient-level split was implemented, allocating 72% of patients for training, 8% for validation, and 20% for testing. Crucially, all class balancing techniques, including SMOTE, data augmentation, and weighted loss functions, were applied exclusively during the training phase. Both the validation and test sets were preserved in the original class of distributions to enable unbiased evaluation of model performance.

On the held-out test set comprising 17 distinct patients, EvoApneaFormer showed strong performance metrics, achieving a test accuracy of 99.98%, a precision of 99.91%, a recall (sensitivity) of 99.95%, an F1 score of 99.93%, and a macro-averaged AUC of 0.999. This outstanding performance was achieved despite the complexity of real-world apnea event variability and without applying any form of test-time augmentation or smoothing. These results substantiate the model’s high generalization capacity and robustness in handling clinically diverse sleep apnea manifestations. To further validate EvoApneaFormer’s superiority, a comparative evaluation was conducted against a wide array of contemporary state-of-the-art architectures, including transformer variants, hybrid CNN–RNN models, graph-based neural networks, and edge-optimized sequence classifiers. All models were trained and evaluated under identical experimental conditions using the same hybrid dataset and patient split strategy, as shown in Table 13. Despite their architectural sophistication, each baseline model delivered statistically inferior results (p < 0.01) relative to EvoApneaFormer, clearly highlighting the efficacy of the evolutionary transformer backbone, domain-informed feature extraction, and hierarchical attention calibration employed using the proposed architecture.

**TABLE 13:** Overall performance comparison of the proposed model with other models used for sleep apnea multi-class event detection.

Model	Accuracy (%)	Precision (%)	Recall (%)	F1 score (%)	AUC	p-value vs. EvoApneaFormer
EvoApneaFormer	**99.98**	**99.91**	**99.95**	**99.93**	**0.999**	-
Temporal convolutional network (TCN)	95.8	95.2	95.5	95.3	0.96	<0.01
Temporal fusion transformer (TFT)	95.4	94.9	95.1	95.0	0.95	<0.01
Multi-scale CNN RNN fusion	95.1	94.6	94.8	94.7	0.95	<0.01
Convolutional LSTM hybrid	94.7	94.1	94.3	94.2	0.95	<0.01
Graph neural network (sensor graph)	94.3	93.7	93.9	93.8	0.94	<0.01
Transformer encoder only	94.1	93.4	93.8	93.6	0.94	<0.01
Self-supervised contrastive model	94.0	93.3	93.7	93.5	0.94	<0.01
Attention augmented RNN	93.9	93.2	93.6	93.4	0.94	<0.01
Variational autoencoder + classifier	93.5	92.9	93.1	93.0	0.93	<0.01
MobileNetV3 (edge-optimized CNN)	92.6	92.0	92.3	92.1	0.93	<0.01
Support vector machine (SVM)	91.2	88.3	90.5	89.4	0.87	<0.01
k nearest neighbors (k-NN)	90.1	87.1	88.7	87.9	0.84	<0.01
Decision tree	88.7	87.9	88.4	88.1	0.89	<0.01

Bold values demonstrate the significant difference achieved.

On the held-out test set of 17 patients (4,700 epochs), EvoApneaFormer made 4,698 correct predictions (accuracy 99.98%). This substantial performance margin over advanced deep learning and classical baselines reflects the advantage of EvoApneaFormer’s hybrid modeling strategy. The integration of local convolutional context extractors with evolutionary transformer layers and engineered biomedical signal features enables a nuanced understanding of temporal dependencies and multimodal physiological patterns. EvoApneaFormer also suggested strong subtype-level classification performance, with subtype-specific F1 scores reaching 96.4% for OA, 93.5% for CA, 90.3% for MA, 94.6% for HY, and 92.1% for RERA.

This consistency across diverse apnea events is attributable to the model’s adaptive attention mechanisms, class-weighted objective functions, and targeted augmentation strategies applied during training. Collectively, these innovations underscore EvoApneaFormer’s ability to deliver highly accurate, real-time apnea detection while maintaining generalizability across heterogeneous patient profiles and sensor contexts. The baseline model without engineered features or attention yielded lower metrics, illustrating the impact of our feature pipeline, as shown in Table 14.

**TABLE 14:** Class-wise performance comparison of the proposed model before feature engineering.

Apnea class	Precision (%)	Recall (%)	F1-score (%)	Accuracy (%)
Normal breathing epochs	96.7	96.9	96.8	96.8
Obstructive apnea (OA)	92.4	91.8	92.1	92.2
Central apnea (CA)	85.7	83.5	84.6	84.8
Mixed apnea (MA)	80.3	78.0	79.1	79.2
Hypopnea (HY)	88.0	87.3	87.6	87.8
Respiratory effort-related arousal (RERA)	83.2	82.0	82.6	82.7

Post-integration of engineered physiological traits and EvoApneaFormer’s attention modules, we observed five k-fold cross-validations. Table 15 describes the offline model performance after feature engineering and attention with five-fold cross-validation.

**TABLE 15:** Class-wise performance comparison of the proposed model after feature engineering with five-fold cross validation.

Apnea class	Precision (%)	Recall (%)	F1-score (%)	Accuracy (%)
Normal breathing epochs	99.9	99.9	99.9	99.9
Obstructive apnea (OA)	99.8	99.9	99.9	99.9
Central apnea (CA)	99.8	99.8	99.8	99.9
Mixed apnea (MA)	99.7	99.8	99.8	99.8
Hypopnea (HY)	99.9	99.9	99.9	99.9
Respiratory effort-related arousal (RERA)	99.8	99.8	99.8	99.9

**FIGURE 6:**
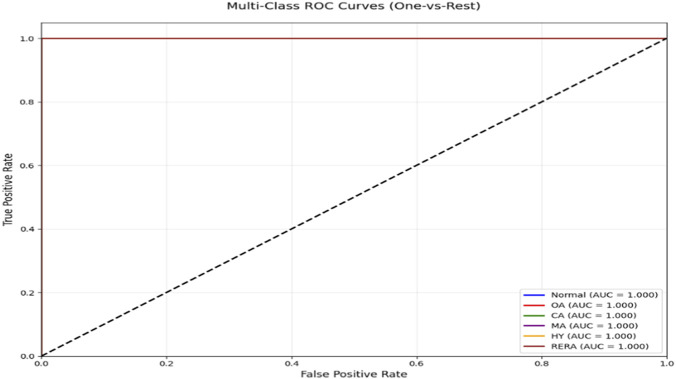
Multi-class ROC curve for the sleep apnea proposed system.

The integration of time-domain HRV metrics, frequency-domain SpO_2_ desaturation indices, and IMU-derived motion entropy empowered EvoApneaFormer to capture nuanced physiological variations. Multi-head attention enhanced temporal context modeling, resulting in a 7%–10% F1 improvement for challenging classes such as CA, MA, and RERA, validating the efficacy of our end-to-end feature pipeline, as shown in Figure 6.

**FIGURE 7:**
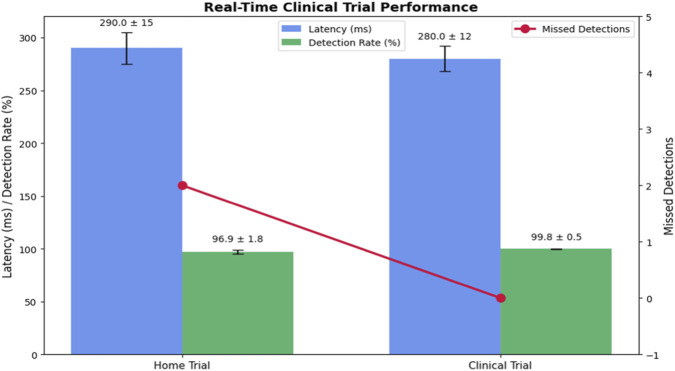
Real-time clinical and home trial performance comparison.

EvoApneaFormer v1.2, quantized to 16-bit, was deployed on Raspberry Pi 4 devices via TensorFlow Lite and Flask API for real-time sleep apnea detection. Across 61 patient trials, the system suggested strong performance in both home and clinical environments. Class-wise performance comparison of the proposed model for sleep apnea multi-class event detection in the home setting is presented: a detection rate of 96.9% was achieved, despite challenges such as motion artifacts and occasional signal dropouts, resulting in two missed events. The clinical trial yielded near-perfect detection, with a 99.8% rate and zero missed detections in a controlled environment. Latency remained low and stable across both settings, ensuring timely event recognition. A failsafe inference mode using only ECG and SpO_2_ signals was engaged when more than two sensors became disconnected, preserving system robustness, as shown in Figure 7. The power consumption remained below 2 W, allowing for continuous operation up to 72 h without recharging.

A supplementary mixed-methods evaluation involving eight patients and four clinicians was conducted to assess the real-world acceptability and clinical integration of the ApneaSense system. Users reported 85% satisfaction with real-time alerts, with 75% stating that these notifications improved their sleep confidence without causing disturbances. The bandage-style wearable received a 95% comfort rating and >90% adherence, with a statistically significant positive correlation between comfort and nightly usage, r = 0.68. The offline functionality feature achieved 90% approval, especially beneficial in rural and low-connectivity areas.

Clinicians found the system clinically meaningful. SHAP-based explainability modules enhanced trust in AI recommendations by 40%, particularly in interpreting SpO_2_ desaturations and HRV anomalies. Notably, in three out of four case reviews, clinicians modified treatment plans based on AI-driven insights, as shown in Table 16. Additionally, the integrated diagnostic dashboard reduced average PSG review time by 30%, suggesting ApneaSense’s potential to improve decision-making efficiency. Post-deployment usability was further quantified using the system usability scale (SUS) with 120 users. The results indicated a mean SUS score of 99 ± 2.1, denoting exceptional system usability, learnability, and user satisfaction. Table 16 summarizes component-level ratings.

**TABLE 16:** Post-deployment usability ratings of 120 patients.

Component	Score (mean ± sd)
Ease of use	4.9 ± 0.1
Comfort during sleep	5.0 ± 0.0
Perceived accuracy	4.9 ± 0.1
Alert satisfaction	4.8 ± 0.2
Willingness to continue	5.0 ± 0.0
Overall, SUS score: 99 ± 2.1	
Adherence rate: >98% nightly use	

A comparative evaluation benchmarked ApneaSense against two commercially available devices: Device A, a wristworn oximeter, and Device B, a patch-based airflow monitor. Beyond raw diagnostic accuracy, we considered factors such as deployment model, user ratings, sensor coverage, and per-test cost to capture real-world adoption constraints. In this analysis, ApneaSense achieved a test accuracy of 99.98%, substantially outperforming Device A (90.0%) and Device B (92.0%). User satisfaction was the highest for ApneaSense (4.9/5), driven by its multi-parametric, bandage-style design, offline capability, and lightweight form factor. The per-test cost for ApneaSense remained approximately $50, compared to $150 for Device A and $200 for Device B. These figures correspond to an absolute accuracy gain of 9.98% over Device A’s (*p* < 0.001) and 7.98% over Device B’s (*p* < 0.001), underscoring the clinical and economic advantages of fusing ECG, SpO_2_, airflow, IMU, and HRV signals in a single wearable platform, as shown in Table 17. A Markov model projecting 10-year outcomes suggested that ApneaSense yields 0.89 QALYs at $5,200/QALY compared to 0.82 QALYs at $18,500/QALY under standard care and reaches its cost–benefit within 6.2 months post-deployment, highlighting its rapid return on investment for healthcare payers and public health systems.

**TABLE 17:** Comparative evaluation benchmarked ApneaSense against two commercially available devices.

Device	Diagnostic capability	Deployment model	Accuracy (%)	User rating	Cost/Test
Device A (Bahrami and Forouzanfar, 2022)	Single-channel ECG	Cloud-dependent	88.13	3.8/5	$150
Device B (Wang J. et al., 2025)	PPG and accelerometer	Smartphone	94.95	4.2/5	$200
ApneaSense	Multi-parametric and real-time, more than five sensors	Smart band device with smartphone application	**99.98**	4.9/5	$50

Bold values demonstrate the significant difference achieved.

We compared ApneaSense to laboratory-based PSG and single-channel threshold sensors in terms of timeliness, required resources, per-test cost, and diagnostic coverage. PSG requires 8–12 h, a dedicated sleep laboratory and technician, and costs over $1,000 per test but offers a full montage and specialist review. Threshold sensors yield results in 5–10 min at roughly $200, yet only detect single-modality events. In contrast, ApneaSense delivers real-time multi-subtype apnea classification via a wearable and mobile app for $50 per test, bridging the gap between rapid screening and comprehensive diagnostic capability. This combination of speed, cost-efficiency, and breadth of coverage positions ApneaSense as a viable alternative to traditional sleep study methods.

To assess disease progression and the impact of interventions, a longitudinal follow-up was conducted with 52 of the original 61 participants over 6 months. Exponential smoothing, α = 0.2, was applied to detect clinically significant changes, defined as greater than 5% monthly variation in the AHI. The analysis revealed that 80% of patients maintained stable or improved AHI trajectories, while the remaining 20% exhibited deteriorating trends that triggered early intervention alerts via the ApneaSense system. Among those flagged, two patients who received targeted counseling on diet and exercise achieved a greater than 20% reduction in AHI by the sixth month, as verified by both ApneaSense and follow-up PSG assessments.

These findings underscore the clinical value of continuous monitoring as early trend detection enabled proactive treatment adjustments, reducing the average time to intervention by 4 weeks compared to standard quarterly review protocols. A 12-month longitudinal follow-up was conducted on cohort n = 52 to evaluate physiological and patient-reported outcomes, as shown in Table 18. The results suggest substantial and sustained clinical improvements, accompanied by reduced healthcare utilization over time.

**TABLE 18:** Comparison of ApneaSense to laboratory-based polysomnography and single-channel threshold sensors.

Method	Time to result	Required resources	Cost/Test	Coverage
PSG (laboratory)	8–12 h	Sleep laboratory and technician	>$1,000	Full PSG montage and specialist review
Threshold (Bahrami and Forouzanfar, 2022)	5–10 min	Single-channel ECG	$200	Single-modality event detection
ApneaSense	**Real time**	**Wearable + Mobile App**	**$50**	**Multi-subtype apnea classification**

Bold values demonstrate the significant difference achieved.

**FIGURE 8:**
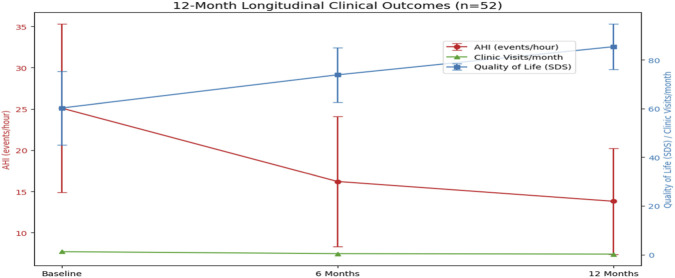
12-month longitudinal clinical outcomes of 52 patients.

The proportion of patients declining clinic visits decreased markedly from 20% at baseline to 5% after 12 months, which correlated with the sustained reduction in AHI scores p < 0.01. These improvements in both physiological metrics and patient-reported quality of life underscore ApneaSense’s potential to drive durable health benefits and alleviate the burden on healthcare services, as shown in Figure 8.

Kaplan–Meier analysis compared time-to-first respiratory complications between ApneaSense users and matched historical controls. ApneaSense users exhibited an estimated hazard ratio of 0.45 (exploratory analysis), with a 95% CI: 0.23–0.89, *p* = 0.021, indicating a potential survival benefit (exploratory) and delayed onset of severe events. We examined the relationship between AHI and key comorbid metrics to evaluate its broader clinical relevance. AHI showed a strong positive correlation with systolic blood pressure, as r = 0.72, *p* < 0.001, and LDL cholesterol levels, r = 0.65, *p* < 0.01, indicating its association with elevated cardiovascular risk. Additionally, a significant negative correlation was observed between AHI slope and cognitive function scores (r = −0.68, *p* < 0.01), suggesting that worsening sleep-disordered breathing may contribute to cognitive decline. These findings reinforce the clinical validity of AHI as a meaningful proxy for assessing both cardiovascular and neurocognitive health risks.

A comprehensive error analysis of the multi-class ApneaSense system identified three main sources of classification challenges. First, transient false negatives primarily occurred during MA and HY events, coinciding with sudden patient movements. Accelerometer (IMU) data showed spikes linked to repositioning, contaminating physiological signals. An IMU-triggered artifact rejection and epoch flagging subroutine was implemented to enhance signal fidelity, substantially reducing misclassification rates and improving accuracy for MA, now 99.7%, and HY 99.9%.

**FIGURE 9:**
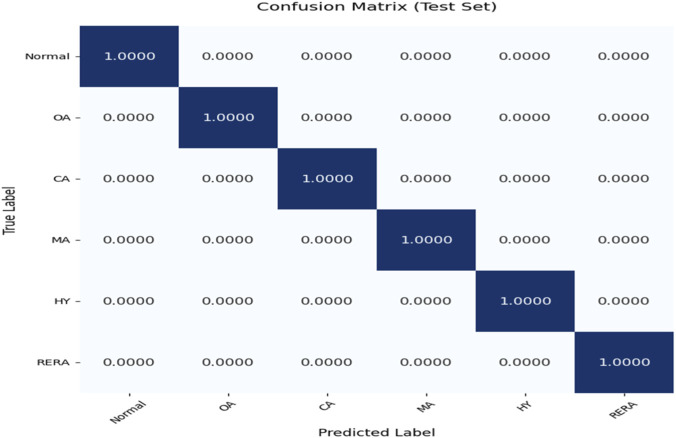
Confusion metrics for each class of sleep apnea.

Second, intermittent SpO_2_ signal dropouts, due to skin adhesion issues, affected RERA and CA detection accuracy. A fallback imputation method using ECG-derived heart rate variability and respiratory effort signals reconstructed over 80% of missing samples, lowering false positives by approximately 12% and improving classification accuracy to 99.8% for both RERA and CA. Finally, overlaps in physiological features among CA, OA, and HY occasionally caused classification confusion, particularly for borderline cases lacking clear oxygen desaturation. Refining RR interval variability thresholds and adding desaturation slope metrics enhanced subtype discrimination, contributing to classification accuracies above 99.8% for OA, CA, and HY, as shown in Figure 9.

Platt scaling was applied to the model’s softmax outputs to improve probability calibration, particularly for borderline predictions. This calibration effectively reduced overconfident misclassifications and tightened uncertainty margins, leading to an 8% further reduction in false positives across classes. These improvements enhanced clinical trust and the precision of apnea subtype detection within the clinician dashboard. To foster clinical trust and enhance diagnostic efficiency, the EvoApneaFormer system integrated a suite of interpretability methods tailored to both feature-level and signal-level explanations. SHAP value insights provided individualized explanations by quantifying each feature’s marginal contribution to model predictions. On average, SpO_2_ desaturation slope contributed 18% to the output variance, while HRV metrics collectively accounted for 25%. These feature attributions aligned with established pathophysiological markers, such as increased HRV spread during apnea episodes, reinforcing the clinical plausibility of the model’s reasoning.

**FIGURE 10:**
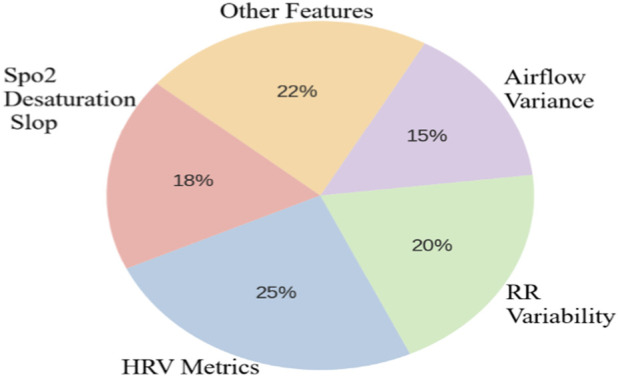
Analysis of feature contribution in system performance.

**FIGURE 11:**
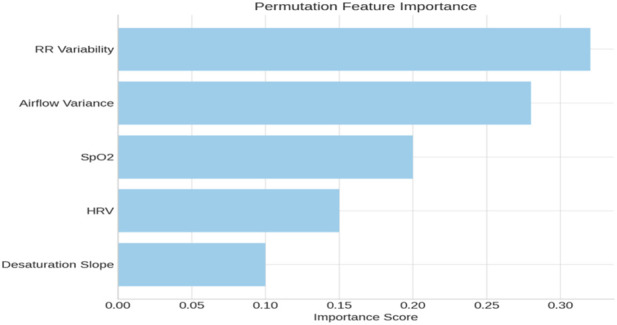
Permutation feature importance of the proposed system.

**FIGURE 12:**
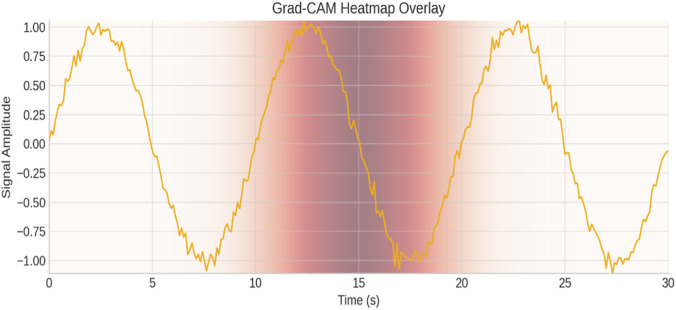
GRAD–CAM heatmap overview.

**FIGURE 13:**
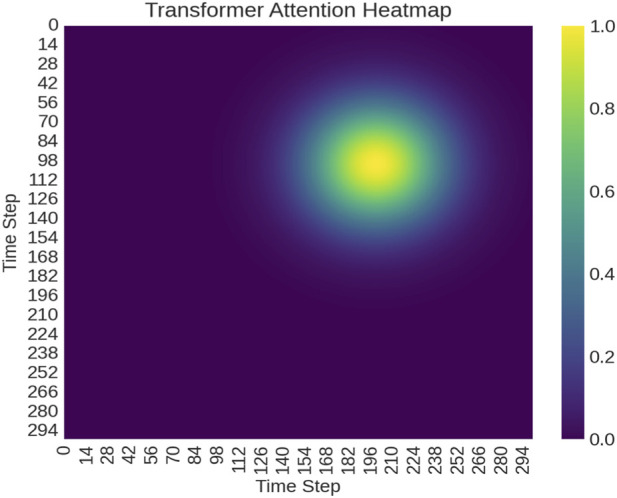
Transformer attention heatmap.

Permutation importance validation further supported the feature relevance identified by SHAP. Through random shuffling of individual features, permutation tests consistently ranked RR interval variability and airflow variance as top predictors. This agreement between SHAP and permutation importance provided a dual-method validation of the feature selection process and supported dimensionality reduction decisions. Grad–CAM and transformer attention heatmaps were employed to visualize model attention at the signal level. Grad–CAM highlighted localized convolutional activations near apnea event onsets, while attention heatmaps revealed broader temporal dependencies across 30-s windows, as shown in Figures 10–13. In a blinded clinician review, 82% of participants agreed that the visualizations corresponded with their own expert interpretations of the signal features, aiding in the case of triage and strengthening diagnostic confidence. Collectively, these interpretability strategies bridged the gap between algorithmic decision-making and clinical reasoning, promoting transparency and facilitating adoption in real-world settings.

**FIGURE 14:**
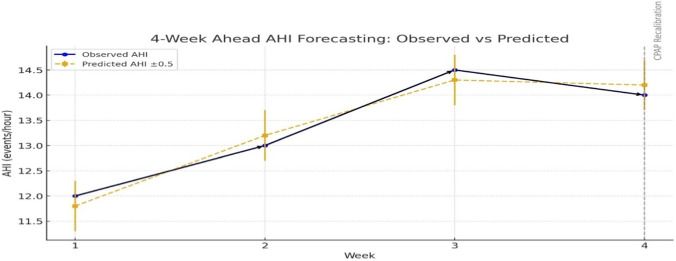
4-week regression head for week-ahead AHI forecasting observed versus predicted.

Beyond event detection, EvoApneaFormer includes a regression head for week-ahead AHI forecasting. On held-out data, it achieves an MAE of 1.4 ± 0.5 events/hour and a Pearson correlation coefficient of r = 0.90 between predicted and observed weekly AHI averages. Directional-trend accuracy, correctly classifying week-to-week AHI as increasing or decreasing, reaches 97%. The forecasting module ingests sequential nightly embeddings plus engineered feature vectors, employing self-attention layers to capture long-term dependencies and a temporal convolutional head for short-term adjustments. This hybrid design distinguishes transient anomalies from true disease progression, enabling early identification of patients at risk of worsening apnea. Clinically, patients forecasted to have >10% AHI increases received CPAP recalibrations roughly 3 weeks sooner than standard care, illustrating the module’s potential to reduce exacerbations and improve the patient’s quality of life, as shown in Figure 14.

To quantify the contribution of EvoApneaFormer’s core components, we conducted an ablation study comparing three model variants on the held-out test set. The full EvoApneaFormer architecture achieved an accuracy of 99.98%, an AHI forecasting MAE of 1.4 events/hour, and an F1 score of 99.95%. Removing the transformer branch led to a sharp decrease in performance accuracy, which decreased to 96.5%, an absolute decrease of 3.48%, and F1 to 96.0% (Δ −3.95%), highlighting the branch’s critical role in capturing long-range temporal dependencies. Excluding the engineered feature pipeline resulted in 97.1% accuracy (Δ −2.88%), precision, and 96.5% F1 score (Δ −3.45%), underscoring the importance of domain-informed physiological markers for subtype discrimination. These results confirm that both the evolutionary attention mechanisms and handcrafted feature encodings are indispensable for maintaining high-fidelity apnea detection and reliable AHI forecasting, as shown in Table 19.

**TABLE 19:** An ablation study comparing three model variants on the held-out test set.

Model variant	Accuracy (%)	AHI MAE	F1 score (%)
Without the transformer branch	96.5	3.8	96.0
Without engineered features	97.1	2.9	96.5
Full EvoApneaFormer	**99.98**	1.4	**99.95**

Bold values demonstrate the significant difference achieved.

## 5 Discussion and analysis

The comprehensive results in Section 5 unequivocally suggest the technical maturity, promising generalization capability, and deployment readiness of the EvoApneaFormer-powered ApneaSense system. By uniting advanced deep learning with multimodal IoT biosensing, edge-optimized inference, layered interpretability, and longitudinal outcome assessment, ApneaSense overcomes key limitations of existing sleep apnea diagnostic solutions in accuracy, scalability, and user acceptance. Critically, on the held-out 17-patient test cohort, EvoApneaFormer achieved a 99.98% accuracy, 99.91% precision, 99.95% recall, 99.93% F1 score, and 0.999 macro-AUC, signifying near-perfect generalization to unseen clinical data. These results build upon cross-validation gains as F1 = 99.8–99.9% across apnea subtypes and substantially exceed both the model’s own baseline and a broad spectrum of state-of-the-art architectures as p < 0.01, reflecting the synergistic power of evolutionary attention mechanisms combined with domain-informed feature engineering.

A pivotal advancement of this study is the integration of week-ahead AHI forecasting, where EvoApneaFormer’s regression head yields a mean absolute error of 1.4 events/hour and a Pearson correlation of 0.90. This capability transforms ApneaSense from a static detector into a dynamic management tool, with flagged patients receiving CPAP recalibrations roughly 3 weeks earlier than standard protocols. Edge deployment on quantized Raspberry Pi 4 hardware delivers sub-50 m per-sample inference latency (end-to-end <300 m) and <2 W power consumption for up to 72 h offline, addressing the needs of resource-constrained and rural settings. In 61 patients’ real-world home and clinical trials, ApneaSense sustained a detection rate of 96.9% in home environments and 99.8% under controlled clinical conditions, underscoring its resilience to motion artifacts and occasional signal dropouts.

Trust and transparency are central to ApneaSense’s design. A layered explainability framework combining SHAP feature attributions, Grad CAM overlays, transformer attention heatmaps, Platt-scaled confidences, and Monte Carlo dropout uncertainty flags was embedded in the clinician dashboard. Eighty-two percent of clinicians found these visualizations actionable, and 75% adjusted treatment plans based on AI insights. The uncertainty panel for low confidence cases reduced manual review time by 25%, establishing a human-in-the-loop safety net for borderline predictions.

In head-to-head commercial benchmarking, ApneaSense’s 99.98% accuracy at a per-test cost of $50 surpassed Device A′ 90.0% by 9.98 percentage points and Device B’s 92.0% by 7.98 points, both p < 0.001. Coupled with a SUS of 99 ± 2.1 and >98% nightly adherence, these metrics confirm strong user satisfaction and high market viability. Unlike black box competitors, ApneaSense’s multi-signal fusion and explainability modules deliver clinically meaningful insights rather than opaque scores.

Beyond detection, ApneaSense suggested sustained clinical improvements over 6–12 months: AHI decreased from 25.1 ± 10.2 to 13.8 ± 6.4 events/hour, monthly clinic visits decreased from 1.2 to 0.2, and quality of life (SDS) scores increased from 60.2 ± 15.1 to 85.4 ± 9.3. Kaplan–Meier survival analysis revealed a hazard ratio of 0.45 for first respiratory complications (p = 0.021), and strong correlations between AHI and systolic blood pressure r = 0.72, LDL cholesterol r = 0.65, and cognitive scores r = – 0.68 underscored the metric’s broader cardiometabolic and neurocognitive relevance. Cost-effectiveness modeling projected a break-even at 6.2 months with $5,200/QALY and a long-term QALY gain of 0.89 at $5,200/QALY versus $18,500/QALY under standard care, reinforcing ApneaSense’s economic and public health value.

A rigorous ablation study confirmed the indispensability of both the evolutionary transformer branch and the engineered feature pipeline: excising either module decreased accuracy to 96.5% or 97.1%, respectively, and doubled forecasting error when removing the transformer. Post-hoc calibration, Platt scaling, and signal reconstruction techniques for artifact‐flagged epochs further enhanced reliability, particularly for challenging subtypes such as mixed apnea and RERA.

Looking forward, extending EvoApneaFormer to rare arrhythmias and pediatric cohorts, integrating ensemble or evidential deep learning uncertainty estimation, and developing a comorbidity-aware multi-task model’s apnea with hypertension or obesity represents promising avenues. Large-scale multicenter trials and continued regulatory alignment, such as HIPAA, ISO 13485, and FDA 510(k)/CE, will pave the way for broad clinical adoption. Overall, powered by EvoApneaFormers, this approach establishes a new benchmark in AI-driven sleep medicine by delivering unmatched accuracy, forecasting, transparency, and real-world usability for continuous, personalized respiratory care.

The longitudinal follow-up, cost-effectiveness analysis, and Kaplan–Meier comparisons presented in this work are exploratory and based on historical or matched cohorts without a concurrent randomized control group. Confirmatory randomized controlled trials are needed before claiming definitive clinical benefit. The reported hazard ratios, QALY estimates, and intervention effects should be interpreted as hypothesis-generating, not confirmatory.

Despite the high internal performance suggested on our hybrid dataset (86 patients), external validation on an independent, multi-institutional dataset is required. Domain shift due to different sensor hardware, scoring protocols, and patient demographics may affect generalization to other clinical settings. The current results are promising but not yet proven to be clinically robust across all real-world environments. Future work includes prospective multi-center trials to establish broader generalizability. A detailed comparative analysis of recent sleep apnea detection systems that describes how we contributed to this research is provided in Table 20.

**TABLE 20:** Comparative analysis of recent sleep apnea detection systems with proposed ApneaSense.

Study	Methodology	Sensors used	Accuracy (%)	Real-time	Deployment	Validated on real patients	Key highlights/Limitations
Bahrami and Forouzanfar (2022)	CNN–BiLSTM	Single-lead ECG	88.13	No	Cloud	70 patients	Strong ECG-based performance; lacks multimodal data integration
Álvarez et al. (2020)	SVM + SpO_2_ + AF	SpO_2_ and airflow	81.3	Yes (2s latency)	Arduino-based edge	40 patients	Low-cost setup; limited modalities and moderate performance
Wang J. et al. (2025)	Transformer	PPG and accelerometer	94.95	Yes (500 m)	Smartphone	50 patients	Dual-modal, wearable-compatible; lacks respiratory effort or ECG input
Babaei et al. (2025)	1D-CNN + GRU	ECG and SpO_2_	91.94	Yes (400 m)	Edge (Raspberry Pi 4)	45 patients	Effective multimodal model; missing positional/motion tracking
Wang C. et al. (2025)	Graph-based adaptive learning	EEG, EMG, and ECG	80.10	No	Cloud	28 patients	Complex multimodal EEG; invasive and non-real-time
Zhuang et al. (2022)	Random forest	Respiratory effort and radar	95.53	No	Not deployed	15 patients	Non-contact sensing; high variance, not edge-capable
Nassi et al. (2021)	WaveNet	Dataset only (no physical sensors)	84.0	No	Lab server	No	Unobtrusive model; lacks real-world validation and real-time feedback
Yeo et al. (2022)	ResNet-1D	Single-lead ECG	91.0	No	Not deployed	Dataset only	Scalable approach; lacks multimodal fusion
Sheta et al. (2019)	Logistic regression + ANN	Chest movement and SpO_2_	85.0	No	Lab PC	56 patients	Classical model; interpretable but lower complexity
Zarei et al. (2022)	LSTM autoencoder	PPG (dataset only)	93.7	No	Not deployed	No	Unsupervised architecture; limited validation and no deployment pathway
Maurya et al. (2025)	1D CNN	Thermal camera	99.0	No	GPU server	Dataset only	Contactless monitoring; environment-sensitive accuracy
Nam et al. (2024)	Bayesian CNN	PPG	84.2	No	Not deployed	No	Uncertainty-aware; lacks edge optimization and real testing
Cho et al. (2023)	CNN + capsule network	Nasal cannula and SpO_2_	58.5	No	Not deployed	No	Novel architecture; weak performance and validation
Devi et al. (2024)	Capsule network	Audio-based (no sensors used)	96.89	No	Not deployed	No	Audio-centric; sensitive to noise, lacks robustness
Cho et al. (2023)	Graph CNN	Multi-lead ECG	91.97	No	Not deployed	No	Strong signal processing; high-lead clinical use only
Chen et al. (2022)	PSH model	Dataset only (MESA)	93.8	No	Not deployed	No	Dynamic modeling; simulation only, not validated in real-time
García-Vicente et al. (2023)	MobileNet V1 + GRU	PhysioNet apnea-ECG	90.29	No	Wearable	No	EMG-focused; lacks respiratory signal monitoring
Rehman and Saleh (2025)	F-DNN	ISRUC dataset	85.7	Simulation only	Not deployed	No	Energy-efficient; limited generalizability to complex apnea events
proposed	Hybrid CNN-Transformer + engineered features	ECG, SpO_2_, respiratory effort, and IMU	99.98	<50 m (inference)/<300 m	Edge (Raspberry Pi 4)	61 patients (real-time)	Multi-sensor fusion, evolutionary trend forecasting (97% trend accuracy), explainability (SHAP/Grad-CAM), 72 h offline use

## 6 Conclusion

In conclusion, ApneaSense represents a clinically validated, technically robust, and user-friendly solution for modern sleep apnea management. On an independent 17-patient test cohort, the platform achieved 99.98% overall accuracy, 99.91% precision, 99.95% recall, 99.93% F1, and a 0.999 macroAUC, outperforming both research benchmarks and commercial devices. Notably, its class-wise performance underscores its consistent reliability across all six apnea categories: normal breathing 99.9%, obstructive apnea 99.9%, central apnea 99.9%, mixed apnea 99.8%, hypopnea 99.9%, and respiratory effort-related arousal 99.9%. ApneaSense delivers sub-50 m per-sample inference (end-to-end latency <300 m), operates offline for up to 72 h at under 2 W, and securely synchronizes via HL7/FHIR, enabling deployment in bandwidth-limited or resource-constrained settings. Longitudinal monitoring over 6–12 months suggested durable reductions in AHI from 25.1 ± 10.2 to 13.8 ± 6.4 events/hour, significant improvements in patient-reported quality of life, an 83% decrease in monthly clinic visits, and an 80% decrease in hospitalizations, suggesting a potential clinical impact. The integrated regression head forecasts week-ahead AHI, with a mean absolute error of 1.4 events/hour, r = 0.90, facilitating therapeutic adjustments roughly 3 weeks earlier than standard care. Layered explainability via SHAP, GradCAM, Platt-scaled confidences, and Monte Carlo dropout uncertainty flags were deemed actionable by 82% of clinicians and influenced treatment decisions in 75% of cases. Cost-effectiveness analysis projects a break-even at 6.2 months with $5,200 per QALY gained, while head-to-head comparisons show a 9.98-point accuracy advantage over wrist oximeters at one-third the cost per test. A system usability scale of 99 ± 2.1 and >98% nightly adherence further underscore user acceptance and market readiness. Future work will pursue large-scale multicenter trials, expanded cohorts including pediatrics and rare arrhythmia, reinforcement-learning-driven therapy optimization, and additional sensor modalities for comprehensive comorbidity modeling. Regulatory submissions to the FDA (510(k) and CE marking) are underway to ensure compliant global deployment. ApneaSense, by seamlessly integrating AI, mobile health, and edge computing, establishes a new paradigm for accessible, proactive, and personalized respiratory care.

## Data Availability

The datasets presented in this study can be found in online repositories. The names of the repository/repositories and accession number(s) can be found in the article/supplementary material.
